# Real-world effectiveness and safety of tenofovir amibufenamide in treatment-naïve chronic hepatitis B: a 48-week retrospective cohort study

**DOI:** 10.3389/fmed.2026.1956363

**Published:** 2026-09-02

**Authors:** Jiarui Zheng, Yuxuan Song, Bo Feng, Yandi Xie

**Affiliations:** Peking University People's Hospital, Peking University Hepatology Institute, Infectious Disease and Hepatology Center of Peking University People's Hospital, Beijing, China

**Keywords:** chronic hepatitis B, functional cure, HBsAg, safety, tenofovir amibufenamide

## Abstract

**Objectives:**

Tenofovir amibufenamide (TMF) is a newly recommended first-line nucleos(t)ide analogues for chronic hepatitis B (CHB) in China. However, real-world evidence on its safety and effectiveness are limited. This study aimed to evaluate the short-term virological efficacy, biochemical response, as well as safety profile of TMF over 48 weeks in a real-world cohort of CHB individuals.

**Methods:**

In this single-center retrospective cohort study, 129 treatment-naïve CHB patients were administered TMF at 25 mg daily. Clinical, virological, and biochemical parameters were gathered at baseline and at weeks 12, 24, and 48 for evaluation. The primary effectiveness endpoint was virological response (VR, HBV DNA <10 IU/mL) after 48 weeks of treatment. Secondary endpoints included VR at other timepoints, alanine aminotransferase (ALT) normalization, and changes in renal parameters and lipid levels.

**Results:**

At week 48, 72.9% (94/129) of patients achieved VR. Multivariate analysis identified lower baseline HBsAg levels, HBeAg negative status and non-cirrhotic status as independent predictors for achieving VR. The ALT normalization rate was 77.5% (100/129) at week 48. One patient achieved HBsAg seroclearance. Regarding safety, no adverse events linked to the medication were observed. There were no statistically significant alterations in serum phosphorus, estimated glomerular filtration rate, serum creatinine, or lipid levels (triglycerides, total cholesterol, high-density lipoprotein and low-density lipoprotein) from baseline to week 48.

**Conclusion:**

In this single-arm observational cohort, TMF treatment achieved effective virological suppression and high rates of ALT normalization by week 48, with renal function and lipid levels remaining stable. Because the study lacked a comparator group, these findings should be regarded as observational. Comparative conclusions about the safety or superiority of TMF relative to tenofovir disoproxil fumarate (TDF) or tenofovir alafenamide (TAF) require dedicated head-to-head studies.

## 1 Introduction

Chronic hepatitis B (CHB) infection remains a main global health issue, significantly endangering human health and acting as a primary cause of advanced liver disease like hepatocellular carcinoma (HCC) and cirrhosis, which are often diagnosed at late stages (1, 2). According to 2022 data, the HBsAg positive prevalence in China reached 5.6%, representing nearly 80 million individuals living with HBV (3). Current management strategies for CHB involves pegylated interferon (PegIFN) alongside nucleos(t)ide analogues (NAs), with the primary goal of preventing the progression to liver cirrhosis and HCC (4, 5). Entecavir, tenofovir alafenamide (TAF), and tenofovir disoproxil fumarate (TDF) are endorsed as initial NAs options given their potent resistance profiles, which effectively suppresses the development of HBV resistance (6–8).

Tenofovir amibufenamide (TMF) is now recommended in mainland China as a first-line NAs for CHB, providing a fourth treatment option (9). Distinguished by a supplementary methyl group versus the prodrug TAF, this structural variation is thought to impart greater stability in the bloodstream and optimize the intracellular conversion process (10). Studies demonstrate that TMF possesses greater stability in plasma than TDF. This property allows it to suppress HBV replication effectively with just 1/30 dose of TDF, thereby achieving a more favorable bone and renal safety profile (11, 12).

Following its recent approval in China, comprehensive evidence from real-world settings on TMF's performance is still lacking. Therefore, this research was conducted to assess the drug's therapeutic impact and safety profile over a 48-week period in CHB patients.

## 2 Materials and methods

### 2.1 Study design and patient selection

This retrospective analysis comprised 156 adults (≥18 years) with documented HBsAg positivity exceeding six months. All participants received care at Peking University People's Hospital from June 2023 through December 2024 and were consecutively enrolled. Exclusion criteria encompassed: (1) coinfection with hepatitis virus C, hepatitis virus D or human immunodeficiency virus, alcohol-related or autoimmune liver disorders, HCC, or pre-chemotherapy antiviral prophylaxis; (2) diagnosed malignancies or other severe conditions compromising survival; (3) introduction or switching of antiviral medications during the observation period; and (4) incomplete medical records. Following screening, 129 eligible participants constituted the final cohort.

The institutional ethics committee at Peking University People's Hospital granted approval for the study protocol (No. 2023PHB117-001). All procedures aligned with the ethical standards established in the 1975 Declaration of Helsinki and its subsequent 1983 amendment. Given the retrospective nature of this investigation, the requirement for individual informed consent was formally waived.

### 2.2 Clinical data collection and definitions

Patient medical information was obtained from our hospital's electronic medical records system. The collected patient characteristics comprised: age, gender, body mass index (BMI), fatty liver disease status, diabetic condition, complete blood count results [including platelet count (PLT)], liver function assessments [aspartate transaminase [AST], alanine aminotransferase [ALT], total bilirubin [TBIL], albumin], renal function evaluations [serum creatinine [Scr], estimated glomerular filtration rate [eGFR]], lipid metabolism indicators [high-density lipoprotein [HDL], low-density lipoprotein [LDL], total cholesterol [TC] and triglycerides], serum phosphorus concentrations, alpha-fetoprotein [AFP] measurements, controlled attenuation parameter [CAP] readings, and liver stiffness measurement [LSM] values. HBV virological and serological markers included HBV DNA, HBsAg and HBeAg. FIB-4 index was computed using the formula: Age (years)×AST (U/L)/PLT (×10^9^/L)×√ALT (U/L). This scoring system serves to determine the severity of hepatic fibrosis, with values meeting or exceeding 3.25 indicating significant liver fibrosis corresponding to Metavir stage F3 or higher, while results below 1.45 effectively exclude the presence of Metavir stage F3 or greater fibrosis. Serum HBsAg and HBV DNA concentrations underwent log_10_ transformation prior to statistical evaluation. HBsAg seroclearance was defined as a quantitative HBsAg level below 0.05 IU/mL. Treatment response was assessed by virological suppression, characterized by HBV DNA levels falling below 10 IU/mL or becoming undetectable. Following 48 weeks of antiviral treatment, low-level viremia (LLV) was identified as either sustained or fluctuating detection of viral DNA maintained below 2,000 IU/mL, with the assay's lower limit of quantification set at 10 IU/mL. Abnormal ALT levels were determined using sex-specific thresholds: values above 30 U/L for male patients and above 19 U/L for female patients. Cirrhosis was defined by clinical, laboratory, and imaging evidence (abdominal ultrasound, CT, MRI, or transient elastography), with LSM cut-offs of 17 kPa (ALT between ULN and 5×ULN and normal bilirubin) and 12 kPa (both ALT and bilirubin within normal ranges) (13); liver biopsy was used when available. All diagnoses were independently confirmed by two hepatologists via medical record review.

### 2.3 Treatment and follow-up

Throughout the investigation period, all newly diagnosed CHB patients without prior treatment were prescribed a daily 25 mg dose of TMF following confirmation of their diagnosis. Adjunctive hepatoprotective medications were administered as clinically indicated based on physician assessment. For all enrolled subjects, clinical outcomes and relevant laboratory measures were systematically obtained over the 48-week monitoring duration. Data collection occurred at predetermined timepoints: initiation of therapy, and subsequently at weeks 12, 24, and 48.

### 2.4 Endpoints

The primary efficacy outcome measured at week 48 was virological response (VR), characterized by plasma HBV DNA concentrations below 10 IU/mL. Secondary outcomes included VR rates during the 12-week and 24-week assessments, along with proportions demonstrating normalized ALT and quantitative HBsAg changes from initial measurements through weeks 12, 24, and 48. Safety endpoints included the change in renal function markers and lipid profiles at weeks 12, 24 and 48 in comparison with the baseline values.

### 2.5 Statistical analysis

Continuous data were presented as mean ± standard deviation or median (interquartile range) and analyzed with Student's t-test and rank sum test. Categorical measures are reported as frequencies with percentages and evaluated using Pearson's chi-square or Fisher's exact probability tests. Logistic regression was used to identify predictors of virological suppression and ALT normalization and variables with *p* *<* *0.05* in univariate logistic regression were entered into the multivariate model. The threshold for statistical significance was established at *p* < 0.05 for all analyses. For missing laboratory measurements at the 12-, 24-, and 48-week time points, if the missing value fell within a predefined window period (±4 weeks), we used the nearest available measurement within that window as a substitute. Values beyond this window were treated as missing and excluded from the analysis at that specific time point. The overall proportion of missing data was <5% across all time points. All computations were performed using R software (version 4.1.1).

## 3 Results

### 3.1 Baseline demographics and clinical features

The final cohort comprised 129 participants who completed the full 48-week follow-up. The study population had an average age of 45.4 years, with male subjects accounting for 68.2% (*n* = 88). Within the cohort, 43 patients (33.3%) were HBeAg-positive, 11 (8.5%) presented with cirrhosis, and 48 (37.2%) were diagnosed with fatty liver disease. The levels of log_10_ HBV DNA and HBsAg (mean ± SD) and ALT [median (IQR)] in HBeAg-negative patients and HBeAg-positive patients were 3.5 ± 1.5 log_10_ IU/mL vs. 7.5 ± 1.7 log_10_IU/mL (*p* < 0.001), 2.9 ± 0.9 log_10_ IU/mL vs. 4.2 ± 0.7 log_10_ IU/mL (*p* < 0.001) and 23.0 (17.0, 37.0) U/L vs. 83.0 (39.0, 148.0) (*p* < 0.001), respectively. The mean creatinine and eGFR levels were 72.0 umol/L and 102.9 mL/min/1.73m^2^, respectively. The mean HDL, LDL, TC and triglycerides levels were 1.4 mmol/L, 2.9 mmol/L, 4.9 mmol/L and 1.2 mmol/L, respectively. The normal ALT ratios were 34.9% (45/129) in overall individuals, 50.0% (43/86) in HBeAg-negative patients and 4.6% (2/43) in HBeAg-positive patients. Table 1 summarized the baseline demographic and clinical features for the complete study population and for subgroups stratified by HBeAg status.

**Table 1:** Baseline clinical characteristics of enrolled patients.

Variables	Total (*n* = 129)	HBeAg-positive (*n* = 43)	HBeAg-negative (*n* = 86)	p
Age (years)	45.4 ± 10.2	40.8 ± 10.1	47.7 ± 9.4	< 0.001
Male (%)	88 (68.2)	34 (79.1)	54 (62.8)	0.061
BMI (kg/m^2^)	24.4 ± 3.7	24.8 ± 3.0	24.3 ± 4.0	0.595
HBV DNA, log_10_ IU/mL	4.8 ± 2.5	7.5 ± 1.7	3.5 ± 1.5	< 0.001
HBsAg, log_10_ IU/mL	3.3 ± 1.0	4.2 ± 0.7	2.9 ± 0.9	< 0.001
PLT (×10^9^/L)	204.0 (172.0, 238.0)	200.0 (171.0, 237.0)	205.0 (177.5, 240.2)	0.529
ALT (U/L)	33.0 (20.0, 63.0)	83.0 (39.0, 148.0)	23.0 (17.0, 37.0)	< 0.001
AST (U/L)	29.0 (23.0, 57.0)	66.0 (31.5, 111.5)	25.0 (21.0, 31.0)	< 0.001
Albumin (g/L)	46.4 ± 4.6	45.8 ± 4.6	46.7 ± 4.5	0.284
TBIL (*μ*mol/L)	16.8 (12.8, 20.3)	17.0 (13.4, 19.9)	16.1 (12.5, 20.4)	0.291
Creatinine (μmol/L)	72.0 ± 13.1	71.7 ± 10.0	72.1 ± 14.5	0.875
eGFR (mL/min/1.73 m^2^)	102.9 ± 13.2	108.2 ± 11.8	100.1 ± 13.1	< 0.001
Phosphorus (mmol/L)	1.1 ± 0.1	1.1 ± 0.2	1.1 ± 0.1	0.975
TC (mmol/L)	4.9 ± 1.0	5.0 ± 1.0	4.8 ± 1.1	0.536
Triglycerides (mmol/L)	1.2 ± 0.6	1.2 ± 0.5	1.2 ± 0.7	0.602
HDL (mmol/L)	1.4 ± 0.3	1.4 ± 0.3	1.4 ± 0.3	0.964
LDL (mmol/L)	2.9 ± 0.8	3.0 ± 0.8	2.9 ± 0.8	0.525
AFP (ng/mL)	3.4 (2.2, 5.4)	4.9 (2.9, 10.6)	2.8 (2.1, 4.3)	0.002
FIB-4	1.3 (0.8, 1.9)	1.5 (0.7, 2.6)	1.2 (0.8, 1.7)	0.318
Fatty liver (%)	48 (37.2)	15 (34.9)	33 (38.4)	0.895
Cirrhosis (%)	11 (8.5)	6 (13.9)	5 (5.9)	0.103
CAP	222.0 (199.0, 258.8)	223.0 (201.2, 243.0)	222.0 (198.8, 264.0)	0.816
LSM (Kpa)	5.5 (4.4, 8.0)	6.1 (4.9, 14.2)	5.2 (4.3, 6.8)	0.045

BMI, body mass index; PLT, platelet; ALT, alanine aminotransferase; AST, aspartate aminotransferase; TBIL, total bilirubin; eGFR, estimated glomerular filtration rate; TC, total cholesterol; HDL, high-density lipoprotein; LDL, low-density lipoprotein; AFP, alpha-fetoprotein; CAP, controlled attenuation parameter; LSM, liver stiffness measurement.

### 3.2 Safety

TMF treatment was well tolerated over the 48-week study duration, with no notable adverse reactions attributed to the medication.

### 3.3 Virological suppression and HBsAg decline

**Figure 1:**
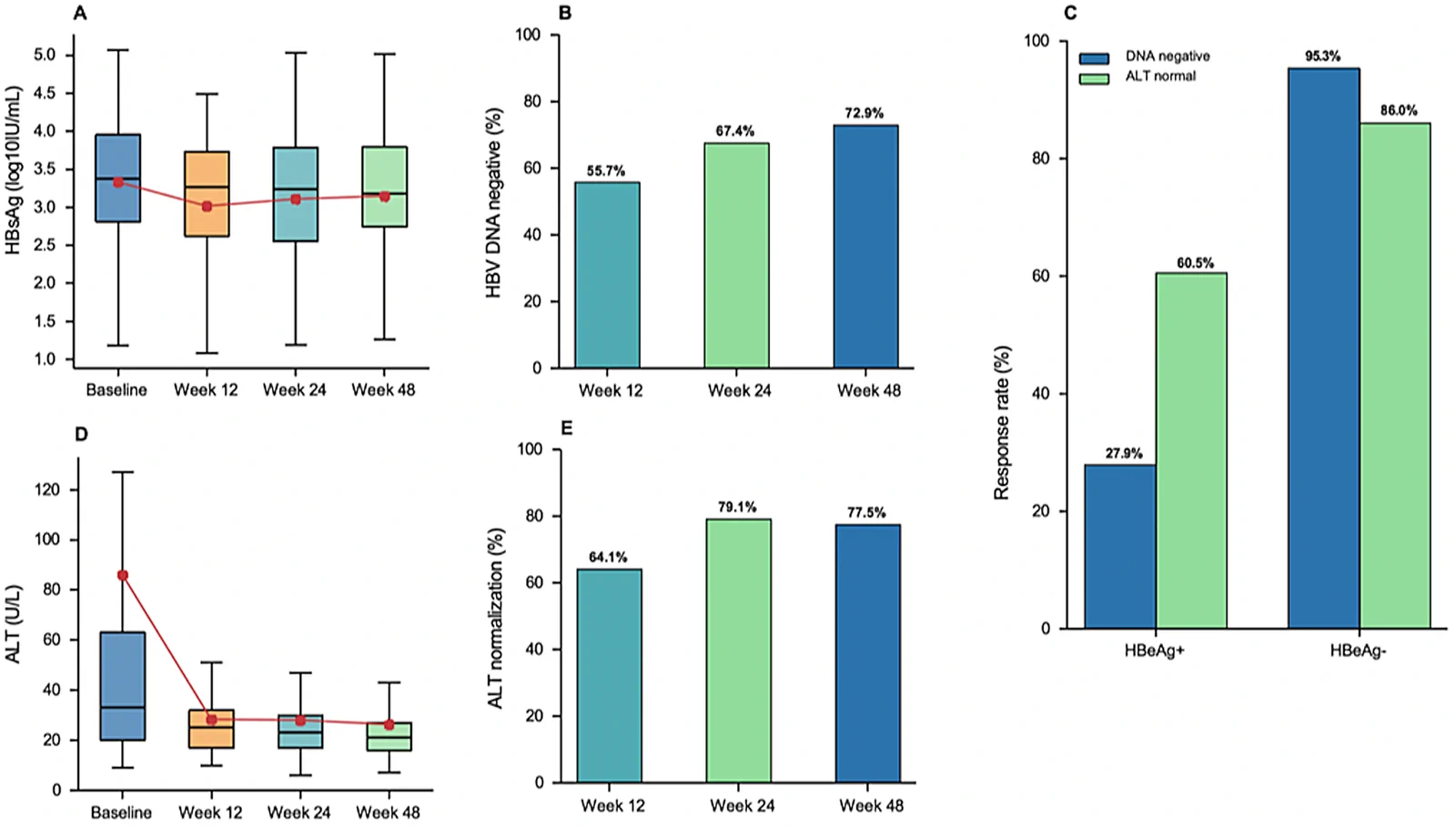
Efficacy overview. **(A)** HBsAg levels at weeks 12, 24 and 48 after treatment. Bars are expressed as mean ± SD; **(B)** HBV DNA suppression rates; **(C)** Week 48 response rates comparing HBeAg + and HBeAg- patients for DNA suppression and ALT normalization; **(D)** ALT levels at weeks 12, 24 and 48 after treatment. Bars are expressed as mean (IQR); **(E)** ALT normalization rates.

The mean level of HBsAg at 12, 24 and 48 weeks were 3.01, 3.11 and 3.15 log_10_IU/mL, respectively, and showed significant difference from baseline (3.33 log_10_IU/mL, *p* < 0.001, Figure 1A). VR was attained by 94 participants (72.9%) at week 48 (Figure 1B). When categorized according to HBeAg status, VR rates demonstrated notable variation: 27.9% among HBeAg-positive individuals compared to 95.3% in HBeAg-negative patients (Figure 1C). LLV was documented in 32 cases (24.8%), constituting 65.1% (28/43) and 4.7% (4/86) of HBeAg-positive and negative subgroups, respectively. Compared with non-responders, subjects attaining VR at week 48 demonstrated older age (46.8 vs. 41.6 years, *p* = 0.01) and reduced frequency of both HBeAg positivity (12.8% vs. 88.6%, *p* < 0.001) and cirrhosis (5.3% vs. 20.0%, *p* = 0.017). Additionally, this group showed substantially lower mean or median baseline values across multiple parameters: HBV DNA (4.1 vs. 6.9 log_10_IU/mL), HBsAg (3.0 vs. 4.2 log_10_IU/mL), ALT (27.0 vs. 49.0 U/L), AST (26.0 vs. 46.0 U/L), eGFR (100.9 vs. 107.9 mL/min/1.73m²), and AFP (3.0 vs. 3.5 ng/mL), with all comparisons having nominal *p* < 0.05 (Table 2). In multivariate logistic regression analysis with variables of *p* *<* 0.05 in univariate analysis, baseline logHBsAg levels [odds ratio [OR] 0.11, 95% confidence interval [CI] 0.03–0.45, *p* = 0.003], HBeAg positive status (OR 0.006, 95% CI 0.001–0.155, *p* = 0.003) and cirrhosis (OR 0.03, 95% CI 0.01–0.49, *p* = 0.017) were independent predictors of VR at week 48 (Table 3). During observational monitoring, a decline of HBsAg ≥0.5 log_10_ occurred in 19 patients (15.1%) and ≥1.0 log_10_ in 10 patients (7.9%). HBsAg seroclearance occurred in only one case, with a baseline qHBsAg level of 0.12 IU/mL, and was first observed at week 12.

**Table 2:** Comparison of baseline characteristics between patients with and without virological suppression at week 48.

Variables	Virological suppression (*n* = 94)	Non-virological suppression (*n* = 35)	*p*
Age (years)	46.8 ± 9.7	41.6 ± 10.6	0.01
Male (%)	62 (66)	26 (74.3)	0.366
BMI (kg/m^2^)	24.4 ± 3.8	24.4 ± 3.4	0.991
HBV DNA, log_10_ IU/mL	4.1 ± 2.1	6.9 ± 2.3	< 0.001
HBsAg, log_10_ IU/mL	3.0 ± 0.9	4.2 ± 0.7	< 0.001
HBeAg positive (%)	12 (12.8)	31 (88.6)	< 0.001
PLT (×10^9^/L)	204.0 (177.5, 236.2)	204.0 (163.5, 240.5)	0.694
ALT (U/L)	27.0 (18.0, 43.5)	49.0 (36.0, 101.0)	< 0.001
AST (U/L)	26.0 (21.2, 34.8)	46.0 (28.5, 72.0)	< 0.001
Albumin (g/L)	46.5 ± 4.2	46.1 ± 5.5	0.698
TBIL (μmol/L)	16.7 (12.5, 20.7)	16.8 (14.2, 19.4)	0.554
Creatinine (μmol/L)	72.8 ± 13.4	70.0 ± 12.3	0.296
eGFR (mL/min/1.73 m^2^)	100.9 ± 13.0	107.9 ± 12.5	0.007
Phosphorus (mmol/L)	1.1 ± 0.1	1.2 ± 0.2	0.704
TC (mmol/L)	4.9 ± 1.0	5.0 ± 1.0	0.615
Triglycerides (mmol/L)	1.2 ± 0.7	1.1 ± 0.5	0.3
HDL (mmol/L)	1.4 ± 0.3	1.4 ± 0.3	0.166
LDL (mmol/L)	2.9 ± 0.8	2.9 ± 0.8	0.847
AFP (ng/mL)	3.0 (2.2, 4.7)	3.5 (2.8, 10.3)	0.075
FIB-4	1.3 (0.9, 1.8)	1.3 (0.6, 2.2)	0.717
Fatty liver (%)	36 (38.3)	13 (37.1)	0.904
Cirrhosis (%)	5 (5.3)	7 (20.0)	0.017
CAP	224.0 (201.2, 264.0)	217.0 (188.8, 236.2)	0.186
LSM (Kpa)	5.3 (4.3, 7.0)	6.2 (5.3, 15.8)	0.049

BMI, body mass index; PLT, platelet; ALT, alanine aminotransferase; AST, aspartate aminotransferase; TBIL, total bilirubin; eGFR, estimated glomerular filtration rate; TC, total cholesterol; HDL, high-density lipoprotein; LDL, low-density lipoprotein; AFP, alpha-fetoprotein; CAP, controlled attenuation parameter; LSM, liver stiffness measurement.

**Table 3:** Summary of the associated factors for virological response at week 48 in univariate and multivariate logistic regression analysis. The multivariate model was adjusted for age, sex, logHBV DNA, logHBsAg, HBeAg status, eGFR and cirrhosis.

Variables	Univariate OR		Multivariate OR	
	95% CI	*p*	95% CI	*p*
Age (years)	1.06 (1.01∼1.1)	0.012	0.95 (0.85∼1.05)	0.324
Male (%)	0.67 (0.28∼1.6)	0.368	1.19 (0.23∼5.73)	0.831
BMI (kg/m^2^)	1 (0.86∼1.16)	0.991		
HBV DNA, log_10_ IU/mL	0.6 (0.49∼0.73)	<0.001	1.66 (0.92∼3.01)	0.096
HBsAg, log_10_ IU/mL	0.12 (0.06∼0.27)	<0.001	0.11 (0.03∼0.45)	0.003
HBeAg positive (%)	0.02 (0.01∼0.06)	<0.001	0.006 (0.001∼0.155)	0.003
PLT (×10^9^/L)	1 (1∼1.01)	0.497		
ALT (U/L)	1 (1∼1)	0.718		
AST (U/L)	1 (1∼1)	0.891		
Albumin (g/L)	1.02 (0.94∼1.1)	0.696		
TBIL (μmol/L)	1 (0.96∼1.04)	0.964		
Creatinine (μmol/L)	1.02 (0.99∼1.05)	0.294		
eGFR (mL/min/1.73 m^2^)	0.95 (0.92∼0.99)	0.011	0.96 (0.89∼1.04)	0.319
Phosphorus (mmol/L)	0.6 (0.04∼8.09)	0.701		
TC (mmol/L)	0.91 (0.62∼1.33)	0.612		
Triglycerides (mmol/L)	1.47 (0.71∼3.06)	0.301		
HDL (mmol/L)	0.38 (0.1∼1.49)	0.167		
LDL (mmol/L)	0.95 (0.59∼1.54)	0.845		
AFP (ng/mL)	0.99 (0.99∼1)	0.151		
FIB-4	0.77 (0.6∼1)	0.106		
Fatty liver (%)	1.05 (0.47∼2.34)	0.904		
Cirrhosis (%)	0.22 (0.07∼0.76)	0.017	0.03 (0.01∼0.49)	0.017

BMI, body mass index; PLT, platelet; ALT, alanine aminotransferase; AST, aspartate aminotransferase; TBIL, total bilirubin; eGFR, estimated glomerular filtration rate; TC, total cholesterol; HDL, high-density lipoprotein; LDL, low-density lipoprotein; AFP, alpha-fetoprotein.

### 3.4 ALT normalization

The median levels of ALT at 12, 24 and 48 weeks were 25, 23 and 21 U/L, respectively, showing statistically significant improvement relative to baseline (33 U/L, *p* < 0.001, Figure 1D). ALT normalization was achieved in 100/129 cases (77.5%) at week 48 (Figure 1E) and HBeAg-negative patients achieved significantly higher rates of ALT normalization (86.0% vs. 60.5%, *p* = 0.002, Figure 1C). Comparative analysis revealed that subjects attaining normal ALT exhibited superior baseline characteristics versus those with sustained elevation (Supplementary Table S1), featuring significantly reduced median values for HBsAg (3.2 vs. 3.8 log_10_IU/mL, *p* = 0.008), ALT (28 vs. 59 U/L, *p* < 0.001), AST (26 vs. 37 U/L, *p* = 0.002), TC (4.8 vs. 5.3 mmol/L, *p* = 0.014), LDL (2.8 vs. 3.2 mmol/L, *p* = 0.026), alongside diminished HBeAg positive prevalence (26.0% vs. 58.6%, *p* = 0.001) and cirrhosis rate (5.0% vs. 24.1%, *p* = 0.005). Multivariable modeling of age, sex and significant univariate predictors (logHBsAg, logHBV DNA, HBeAg status, TC, LDL and cirrhosis) identified HBeAg positive status (OR 0.03, 95% CI 0.01–0.81, *p* = 0.040) and cirrhosis (OR 0.03, 95% CI 0.01–0.40, *p* = 0.009) as independent predictors of ALT normalization at week 48 (Table 4).

**Table 4:** Summary of the associated factors for ALT normalization at week 48 in univariate and multivariate logistic regression analysis. The multivariate model was adjusted for age, sex, logHBV DNA, logHBsAg, HBeAg status, TC, LDL and cirrhosis.

Variables	Univariate OR		Multivariate OR	
	95% CI	*p*	95% CI	*p*
Age (years)	1.01 (0.97∼1.05)	0.763	0.98 (0.91∼1.05)	0.555
Male (%)	1.17 (0.49∼2.81)	0.723	1.51 (0.49∼4.62)	0.472
BMI (kg/m^2^)	0.96 (0.83∼1.11)	0.59		
HBV DNA, log_10_ IU/mL	0.87 (0.73∼1.02)	0.09	1.76 (0.86∼2.98)	0.098
HBsAg, log_10_ IU/mL	0.52 (0.31∼0.85)	0.01	0.57 (0.24∼1.32)	0.191
HBeAg positive (%)	0.25 (0.1∼0.59)	0.002	0.03 (0.01∼0.81)	0.040
PLT (×10^9^/L)	1 (0.99∼1.01)	0.835		
ALT (U/L)	1 (1∼1)	0.784		
AST (U/L)	1 (1∼1.01)	0.674		
Albumin (g/L)	0.93 (0.83∼1.05)	0.268		
TBIL (μmol/L)	1 (0.96∼1.04)	0.815		
Creatinine (μmol/L)	1.01 (0.98∼1.04)	0.603		
eGFR (mL/min/1.73 m^2^)	1 (0.97∼1.03)	0.959		
Phosphorus (mmol/L)	0.81 (0.05∼13.03)	0.881		
TC (mmol/L)	0.6 (0.39∼0.92)	0.018	0.22 (0.02∼2.09)	0.192
Triglycerides (mmol/L)	0.62 (0.33∼1.15)	0.13		
HDL (mmol/L)	0.59 (0.14∼2.43)	0.464		
LDL (mmol/L)	0.56 (0.33∼0.94)	0.029	0.91 (0.10∼8.54)	0.937
AFP (ng/mL)	1 (0.99∼1.01)	0.507		
FIB-4	0.9 (0.7∼1.15)	0.398		
Fatty liver (%)	0.48 (0.21∼1.11)	0.087		
Cirrhosis (%)	0.17 (0.05∼0.57)	0.004	0.03 (0.01∼0.40)	0.009

BMI, body mass index; PLT, platelet; ALT, alanine aminotransferase; AST, aspartate aminotransferase; TBIL, total bilirubin; eGFR, estimated glomerular filtration rate; TC, total cholesterol; HDL, high-density lipoprotein; LDL, low-density lipoprotein; AFP, alpha-fetoprotein;.

### 3.5 Changes in renal function

**Figure 2:**
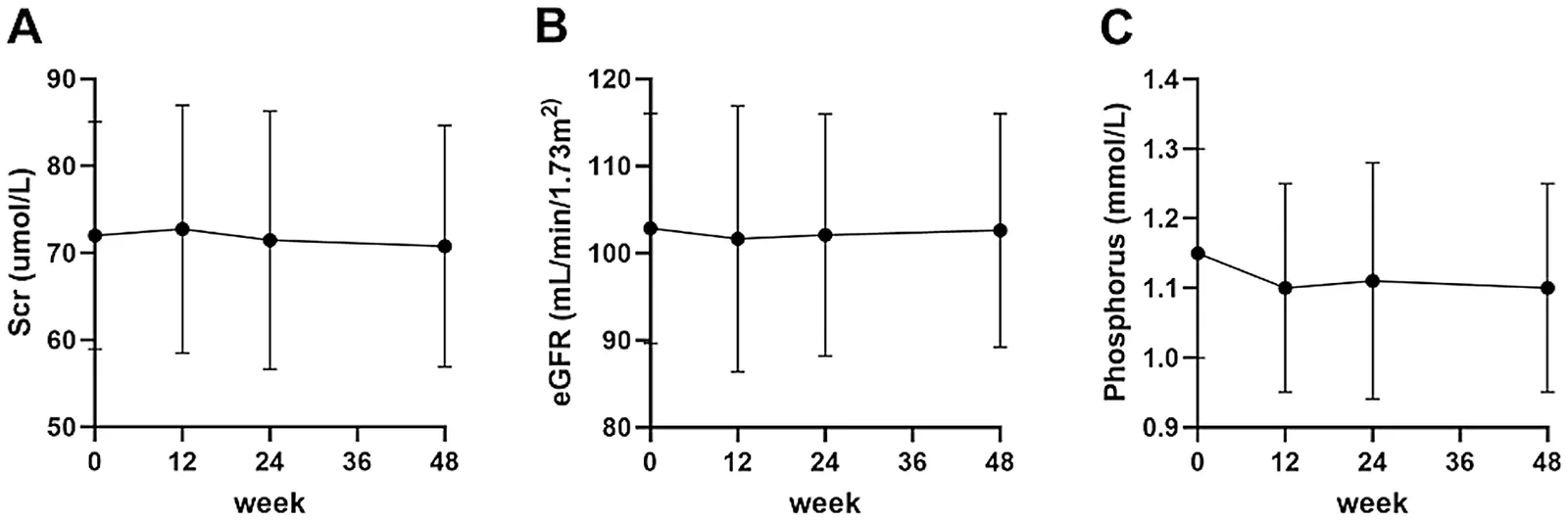
Renal safety profiles at weeks 12, 24 and 48 after treatment. **(A)** Scr levels. Bars are expressed as mean ± SD. **(B)** eGFR levels. Bars are expressed as mean ± SD. **(C)** Phosphorus levels. Bars are expressed as mean ± SD.

After 12, 24 and 48 weeks of treatment, Serum creatine (Scr) levels varied from 72.0 μmol/L to 72.7 μmol/L, 71.5 μmol/L and 70.8 μmol/L, respectively (Figure 2A). Meanwhile, eGFR levels varied from 102.9 mL/min/1.73m^2^ to 101.6 mL/min/1.73m^2^, 102.1 mL/min/1.73m^2^ and 102.6 mL/min/1.73 m^2^, respectively (Figure 2B), and phosphorus levels varied from 1.15 mmol/L, 1.10 mmol/L, 1.11 mmol/L and 1.10 mmol/L, respectively (Figure 2C). Nevertheless, no significant differences were observed in Scr, eGFR and phosphorus within 48 weeks.

### 3.6 Changes in blood lipids

**Figure 3:**
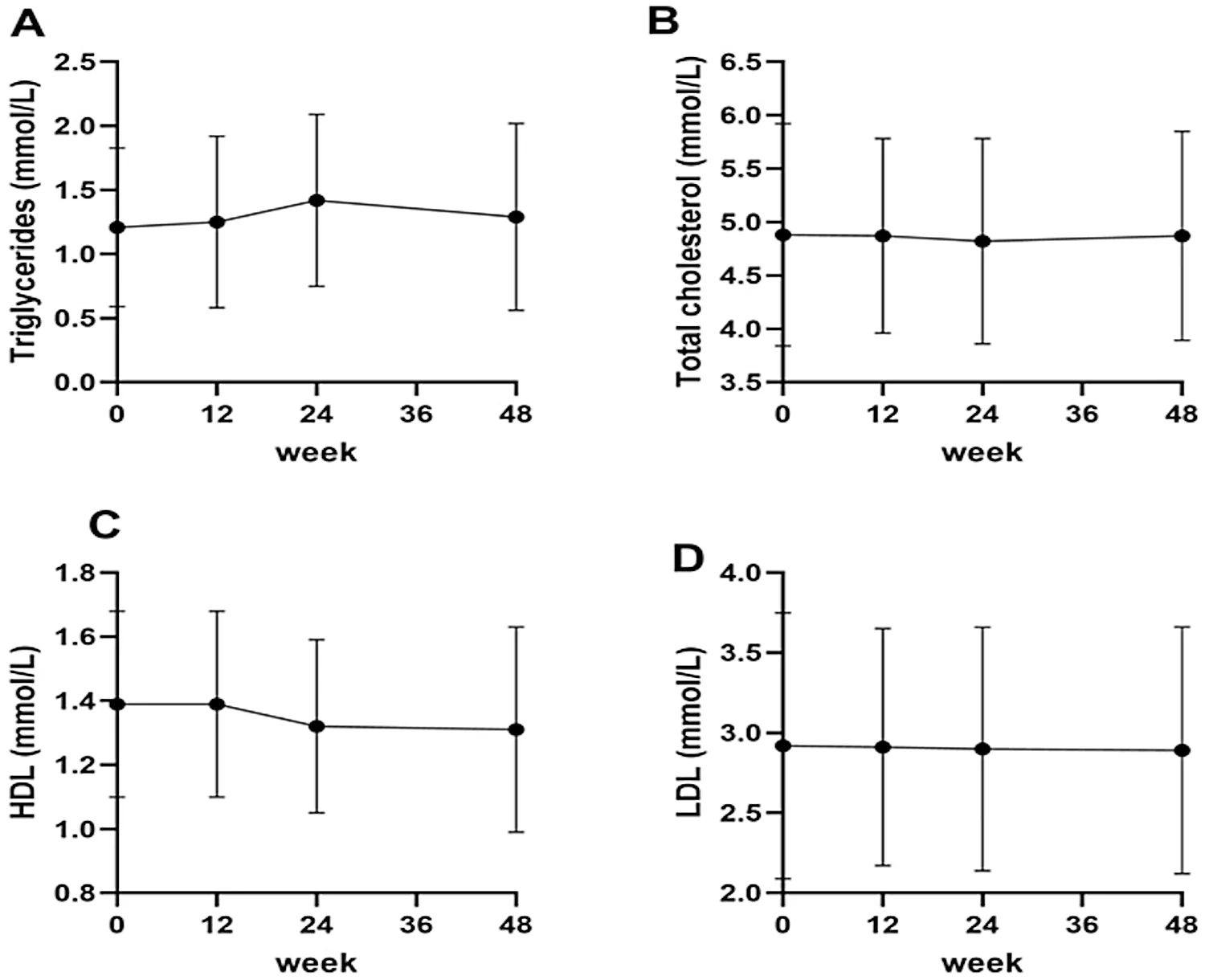
Blood lipid profiles at weeks 12, 24 and 48 after treatment. **(A)** Triglycerides levels. Bars are expressed as mean ± SD. **(B)** Total cholesterol levels. Bars are expressed as mean ± SD. **(C)** HDL levels. Bars are expressed as mean ± SD. **(D)** LDL levels. Bars are expressed as mean ± SD.

The present investigation evaluated key lipid parameters—triglyceride, TC, HDL and LDL and no significant alteration was observed at 12, 24 and 48 weeks compared with baseline (Figures 3A–D).

## 4 Discussion

In our study of patients under real-world conditions, 48-week TMF monotherapy for CHB resulted in a 0.2 log_10_IU/mL decline in HBsAg. Virological suppression was achieved by 72.9% of participants, while 77.5% (100/129) showed normalized ALT levels. Regarding safety, markers of renal function and blood lipid profiles showed no significant changes over the observation period.

Our investigation revealed that TMF therapy resulted in a 67.4% VR rate following 24 weeks of treatment and further increased to 72.9% at week 48, indicating a time-dependent enhancement of antiviral efficacy, and HBeAg-negative patients achieving a significantly higher VR rate than HBeAg-positive patients (95.3% vs. 27.9%). In a retrospective study, 78.6% CHB patients achieved VR (HBV DNA < 10IU/mL) after 48 weeks of TMF treatment (14). In a multicenter, randomized clinical trial, in the HBeAg-negative population, 88.9% achieved HBV DNA less than 20 IU/mL in the TMF group after 48-week treatment (15). In a multicenter, prospective, real-world cohort study, VR (HBV DNA < 20 IU/mL) rate was 42.86% and 90.48% for TMF at 24 and 48 weeks, respectively (16). This discrepancy may be attributed to the different lower limits of threshold used to define VR, as our study applied a more stringent threshold of HBV DNA < 10 IU/mL. Besides, it is important to note that although patients were advised to take TMF with meals, actual adherence to this instruction could not be verified retrospectively and given the pharmacokinetic evidence that a high fat meal significantly increases drug absorption, nonstandard administration may have partially contributed to the different response rate observed in our study. Furthermore, this study found that 24.8% of TMF-naïve individuals had LLV following 48-week therapy, with HBeAg-positive patients accounting for 65.1%, which had a higher prevalence than HBeAg-negative individuals (4.7%). This finding holds important clinical significance because persistent LLV correlates with advancement of chronic hepatic pathology (17, 18).

The relatively low VR rate (27.9%) was observed in HBeAg-positive patients at week 48 in our study. Several factors may account for this finding. First, we applied a stringent threshold for VR (HBV DNA <10 IU/mL). When using the commonly used cutoffs of <20 IU/mL or <100 IU/mL in many prior studies, the VR rates in HBeAg-positive patients increased to 37.2% and 53.5%, respectively. Second, the HBeAg-positive subgroup had a markedly higher baseline viral load (mean 7.5 log10 IU/mL) compared with the HBeAg-negative group, and high baseline viraemia is a well-established predictor of slower VR to NAs (19, 20). Third, the 48-week treatment duration may be insufficient for complete VR in patients with high baseline viral loads and positive HBeAg status, and longer follow-up may be necessary to assess delayed responses (21). These observations underscore the importance of baseline risk stratification and the need for individualized treatment strategies in this patient population.

The persistent nature of covalently closed circular DNA (cccDNA) in hepatocytes poses a major barrier to viral eradication, making functional cure the principal objective in CHB management (4, 22, 23). Despite effective viral suppression with NAs, reducing HBsAg levels or achieving seroclearance remains challenging (1, 24, 25). A comprehensive multicenter investigation monitoring 4,769 CHB subjects receiving entecavir or tenofovir therapy over 26,614 person-years documented merely 2% cumulative HBsAg clearance after ten years (26). Similarly, an analysis of 5,409 patients treated with entecavir or lamivudine revealed even lower seroclearance rates, with an annual incidence of 0.3% (27). The results of our study were comparable to those of the previous studies. While quantitative HBsAg measurements demonstrated a gradual decline, this reduction occurred at an insufficient rate to facilitate seroclearance. Among the 129 individuals receiving TMF therapy, a single case attained HBsAg seroclearance (HBsAg < 0.05 IU/mL), though this subject's baseline qHBsAg measured merely 0.12 IU/mL. This outcome underscored that HBsAg clearance or seroconversion remains challenging with TMF, similar to other oral anti-HBV agents.

Multivariable analysis showed that baseline logHBsAg levels (OR 0.11, 95% CI 0.03–0.45, *p* = 0.003), HBeAg positive status (OR 0.006, 95% CI 0.001–0.155, *p* = 0.003) and liver cirrhosis (OR 0.03, 95% CI 0.01–0.49, *p* = 0.017) as significant determinants of VR at week 48. These findings suggest enhanced likelihood of achieving VR among TMF-treated patients presenting with reduced initial HBsAg concentrations, HBeAg negative status and absence of cirrhosis, which were consistent with prior evidence that lower baseline HBsAg levels have been independently associated with higher rates of VR (28), while HBeAg positivity and presence of cirrhosis were consistently identified as negative predictors of treatment outcome (28, 29).

Additionally, we found that ALT level significantly decreased after TMF treatment as confirmed by many previous studies (30, 31). The overall ALT normalization rate at week 48 in our cohort was 77.5%, which is lower than the rates of approximately 90% reported in previous studies (32, 33). A possible explanation is that those studies used ALT <40 U/L as the upper limit of normal, whereas our study adopted a more stringent cutoff value (above 30 U/L for male patients and above 19 U/L for female patient). Normal on-treatment ALT was associated with a lower risk of hepatic events in patients receiving NA treatment (34). Multivariate analysis identified HBeAg status and cirrhosis were significant factors influencing ALT normalization at week 48. HBeAg-positive patients (OR 0.03, 95% CI 0.01–0.81, *p* = 0.03) and those with cirrhosis (OR 0.03, 95% CI 0.01–0.40, *p* = 0.009) had significantly lower rates of ALT normalization, indicating that these patients may require longer treatment duration or more intensive therapeutic strategies to fully benefit.

Nephrotoxicity represents a well-documented complication of tenofovir-based therapies. Previous comparative studies have established TAF's superior renal safety performance relative to TDF (33, 35, 36). Our real-world investigation, consistent with existing literature, observed no significant renal impairment associated with TMF administration (14, 31). The favorable nephrotoxicity profile of TMF was likely attributed to its unique pharmacokinetic characteristic, which incorporated a methyl group and enhanced its stability in plasma and reduced drug exposure in renal tissues. This targeted delivery mechanism allowed TMF to achieve similar intracellular concentrations in hepatocytes at a significantly lower dose, while substantially reducing drug accumulation in the kidneys and bones (11, 16).

For individuals undergoing prolonged antiviral regimens for CHB, maintaining stable lipid profiles is a critical clinical concern (37). The cumulative metabolic consequences of these medications significantly influence patients' overall cardiovascular health and quality of life over time. This analysis revealed that a 48-week TMF regimen did not induce statistically significant alterations in key lipid parameters, including TG, TC, LDL and HDL. Randomized comparisons have demonstrated that TDF-based regimens are generally associated with lower lipid levels, whereas TAF is associated with modest increases in some lipid parameters (33, 35). The lipid changes observed with TMF are consistent with reports from real-world TMF cohorts (16) and with a TDF-to-TMF switch analysis (37). Because our study lacked a comparator group, these observations should be regarded as descriptive.

While the study has certain limitations, such as a small sample, relatively short-term monitoring, absence of comparator groups (e.g., TDF/TAF), selection bias from its retrospective approach and the inability to verify medication adherence or food intake retrospectively, its results still affirmed TMF's important place in CHB therapy. Because the study lacked a comparator arm, these findings should not be interpreted as evidence of superiority or of absolute safety relative to other agents.

## 5 Conclusion

In summary, in this single-arm observational cohort, TMF showed effective antiviral suppression, favorable biochemical improvement, stable renal function and lipid metabolism in the treatment of CHB over 48 weeks. Because the study lacked a comparator group, these findings are observational and do not establish the relative safety or superiority of TMF compared with TDF or TAF. Longer-term, prospective, comparative studies are needed to further clarify the value of TMF in the comprehensive management of CHB.

## Data Availability

The raw data supporting the conclusions of this article will be made available by the authors, without undue reservation.

## Supplementary material

SUPPLEMENTARY TABLE S1 Comparison of baseline characteristics between patients with and without ALT normalization at week 48.
