# Supplementary material for: Real-world effectiveness and safety of tenofovir amibufenamide in treatment-naïve chronic hepatitis B: a 48-week retrospective cohort study
Source: Front Med (Lausanne). 2026 Sep 2;13:1956363. doi: 10.3389/fmed.2026.1956363 (PMC13577848; doi:10.3389/fmed.2026.1956363)
Supplement: Supplementary file 1 [file Table1.docx]

| Variables | ALT normalization (n = 100) | Non-ALT normalization (n = 29) | *p* |
| --- | --- | --- | --- |
| Age (years) | 45.5 ± 9.4 | 44.9 ± 12.7 | 0.765 |
| Male (%) | 69 (69) | 19 (65.5) | 0.723 |
| BMI (kg/m^2^) | 24.3 ± 2.9 | 24.8 ± 5.0 | 0.595 |
| HBV DNA, log_10_ IU/mL | 4.6 ± 2.4 | 5.5 ± 2.8 | 0.088 |
| HBsAg, log_10_ IU/mL | 3.2 ± 1.0 | 3.8 ± 1.0 | 0.008 |
| HBeAg positive (%) | 26 (26.0) | 17 (58.6) | 0.001 |
| PLT (× 10^9^/L) | 204.5 (172.2, 236.8) | 203.0 (165.0, 257.0) | 0.879 |
| ALT (U/L) | 28.0 (18.0, 44.0) | 59.0 (37.0, 96.0) | < 0.001 |
| AST (U/L) | 26.0 (21.8, 42.2) | 37.0 (28.0, 74.0) | 0.002 |
| Albumin (g/L) | 46.1 ± 5.0 | 47.2 ± 2.9 | 0.269 |
| TBIL (μmol/L) | 17.0 (12.5, 20.2) | 16.6 (13.8, 20.8) | 0.572 |
| Creatinine (μmol/L) | 72.3 ± 13.1 | 70.9 ± 13.3 | 0.607 |
| eGFR (ml/min/1.73 m^2^) | 102.9 ± 11.6 | 102.8 ± 17.8 | 0.959 |
| Phosphorus (mmol/L) | 1.1 ± 0.1 | 1.1 ± 0.2 | 0.883 |
| TC (mmol/L) | 4.8 ± 1.0 | 5.3 ± 1.1 | 0.014 |
| Triglycerides (mmol/L) | 1.2 ± 0.6 | 1.4 ± 0.8 | 0.12 |
| HDL (mmol/L) | 1.4 ± 0.3 | 1.4 ± 0.3 | 0.467 |
| LDL (mmol/L) | 2.8 ± 0.8 | 3.2 ± 0.8 | 0.026 |
| AFP (ng/mL) | 3.0 (2.3, 4.9) | 3.7 (2.1, 6.1) | 0.393 |
| FIB-4 | 1.3 (0.8, 1.8) | 1.1 (0.7, 2.0) | 0.636 |
| Fatty liver (%) | 34 (34) | 15 (51.7) | 0.083 |
| Cirrhosis | 5 (5.0) | 7 (24.1) | 0.005 |
| CAP | 219.5 (199.0, 245.8) | 228.5 (200.5, 276.0) | 0.43 |
| LSM (Kpa) | 5.2 (4.3, 7.0) | 6.2 (5.4, 13.6) | 0.072 |

**TABLE S1. Comparison of baseline characteristics between patients with and without ALT normalization at week 48.** BMI, body mass index; PLT, platelet; ALT, alanine aminotransferase; AST, aspartate aminotransferase; TBIL, total bilirubin; eGFR, estimated glomerular filtration rate; TC, total cholesterol; HDL, high-density lipoprotein; LDL, low-density lipoprotein; AFP, alpha-fetoprotein; CAP, controlled attenuation parameter; LSM, liver stiffness measurement.
